# Autologous semitendinosus meniscus graft significantly improves knee joint kinematics and the tibiofemoral contact after complete lateral meniscectomy

**DOI:** 10.1007/s00167-022-07300-z

**Published:** 2023-01-05

**Authors:** Andreas M. Seitz, Janina Leiprecht, Jonas Schwer, Anita Ignatius, Heiko Reichel, Thomas Kappe

**Affiliations:** 1 Institute of Orthopedic Research and Biomechanics, Center of Trauma Research Ulm, University Medical Center, Ulm University, Helmholtzstrasse 14, 89081 Ulm, Germany; 2 Department of Orthopedic Surgery, RKU, Ulm University Medical Center, Ulm, Germany

**Keywords:** Lateral meniscus, Tendon autograft, Total replacement, Meniscectomy, Tibiofemoral contact, Kinematics

## Abstract

**Purpose:**

The purpose of this study was to investigate the potential of a doubled semitendinosus (ST) and a single gracilis tendon (GT) lateral meniscus autograft to restore the knee joint kinematics and tibiofemoral contact after total lateral meniscectomy (LMM).

**Methods:**

Fourteen human knee joints were tested intact, after LMM and after ST and GT meniscus autograft treatment under an axial load of 200 N during full range of motion (0°–120°) and four randomised loading situations: without external moments, external rotation, valgus stress and a combination of external rotation and valgus stress using a knee joint simulator. Non-parametric statistical analyses were performed on joint kinematics and on the tibiofemoral contact mechanics.

**Results:**

LMM led to significant rotational instability of the knee joints (*p* < 0.02), which was significantly improved after ST autograft application (*p* < 0.04), except for knee joint flexions > 60°. The GT autograft failed to restore the joint kinematics. LMM significantly increased the tibiofemoral contact pressure (*p* < 0.03), while decreasing the contact area (*p* < 0.05). The ST autograft was able to restore the contact mechanics after LMM (*p* < 0.02), while the GT replacement displayed only an improvement trend.

**Conclusion:**

The doubled ST lateral meniscus autograft improved the knee joint kinematics significantly and restored the tibiofemoral contact mechanics almost comparable to the native situation. Thus, from a biomechanical point of view, ST meniscus autografts might be a potential treatment alternative for patients who are indicated for meniscus allograft transplantation.

**Supplementary Information:**

The online version contains supplementary material available at 10.1007/s00167-022-07300-z.

## Introduction

Meniscal injuries are among the most common injuries within the knee joint [3, 10]. Dependent on the localisation and type of meniscus injury, detrimental arthroscopic meniscectomy procedures cannot always be avoided [25]. To overcome the devastating meniscectomy effects and delay the onset of osteoarthritis (OA), several options have, to date, been developed for total meniscus replacement. The current artificial treatment options focus on biomechanically adequate placeholders, like the polyurethane-based NUsurface (Active Implants LLC, Memphis, TN, USA) [5] and the Trammpolin (Atro Medical, Nijmegen, Netherlands) [41] meniscus prostheses. Alternatively, total replacement can be performed using allogenic transplants. Such meniscus allograft transplants (MAT) provide the best possible morphological characteristics and biomechanical properties when attempting to replace the resected meniscus [31]. By contrast, MAT are also associated with administrative, cost, storage and legal issues, particularly for fresh or fresh frozen implants [32]. Additionally, high rates of biomechanical insufficiency arising from size mismatching, secondary degeneration, poor availability and high re-surgery rates have been reported [36, 39]. As an MAT alternative, autologous substitutes for meniscal replacement were previously investigated with the aim to provide a geometrically adaptable three-dimensional scaffold that enhances endogenous cell ingrowth. Autologous substitutes are advantageous because of their availability, biocompatibility and remodelling potential. Fat pads [18], perichondral tissue [2], fascia-covered meniscus fragments [15, 16], and tendons [13, 17, 22] have been used in small and large animal models and these all demonstrated a chondroprotective effect of the autograft, with a remodelling trend of transplanted tendons towards meniscus-like tissue. Despite these promising results, the literature lacks of studies regarding the impact of an autologous tendon meniscus autograft on the knee joint biomechanics. Therefore, the purpose of this study was to investigate the potential of a doubled semitendinosus (ST) and a single gracilis tendon (GT) lateral meniscus autograft to restore the knee joint kinematics and tibiofemoral contact after total lateral meniscectomy (LMM). On the basis of the positive outcomes of the animal and clinical studies, it was hypothesised that a doubled ST autograft is biomechanically able to sufficiently substitute the lateral meniscus with a concomitant similar tibiofemoral load distribution and joint kinematics compared to the native meniscus state. In case the hypothesis would be confirmed, an autologous tendon meniscus replacement might be, from a biomechanical point of view a viable alternative to MAT.

## Materials and methods

**Fig. 1:**
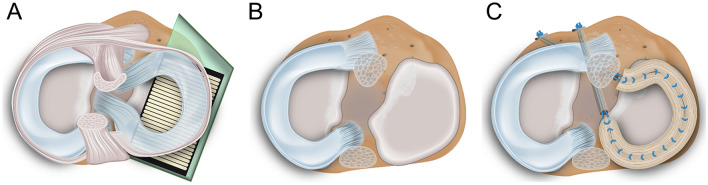
Schematic representation of **A** a native knee joint with inserted pressure mapping sensors (Sensor 4000, sensitivity 10,324 kPa; Tekscan Inc., Norwood, MA, USA) on the tibial lateral compartment; **B** total lateral meniscectomy; **C** total meniscus replacement using a doubled semitendinosus tendon autograft and a single-bundle gracilis tendon autograft (not visualised)

Following IRB approval (No. 37/20; University of Ulm), fourteen non-osteoarthritic fresh frozen cadaveric knees (11 males, 3 females; all left knees; median age 57 years, range 28–64 years; Science Care, Phoenix, AZ, USA) were thawed at room temperature and both the gracilis tendon (GT) and ST were harvested following a standard clinical protocol [43]. The GT was shortened to a total length of 14 cm and armed with sutures at both ends (FiberWire II, Arthrex Inc., Naples, FL, USA). The ST was shortened to 24–28 cm, doubled over a total length of 14 cm and both strings were sewed together. The autografts were armed at both ends by means of a modified Mason-Allen stitch pull-out repair using FiberWire (FiberWire II, Arthrex Inc.) as previously described be Lee et al. for lateral meniscus posterior root repair [20]. The cross-sectional area of the tendons was determined using a customised area measurement device (measurement accuracy 0.05 mm^2^) at the three positions representing the midportion of the anterior horn, pars intermedia and posterior horn. The doubled ST meniscus autograft displayed a mean cross-sectional area of 26.7 ± 5.3 mm^2^ and the GT autograft of 12.2 ± 3.4 mm^2^. Subsequently, the joints were dissected, leaving the capsuloligamentous apparatus intact. Diaphyseal transection of the femur and tibia was performed 15 cm proximal and distal from the joint line. For joint stabilisation, the proximal fibula was cut to a length of 5 cm and fixed to the tibia using a cortical screw. Both diaphyses were embedded into cylindrical metal pots using polymethyl-methacrylate (Technovit 3040; Heraeus Kulzer GmbH, Wertheim, Germany). Lateral arthrotomy was performed by anterior and posterior transversal incisions to introduce equilibrated and calibrated (calibration accuracy based on 2nd order polynominal fit: ± 0.5%) pressure mapping sensors (Sensor 4000, sensitivity 10,342 kPa; Tekscan Inc., Norwood, MA, USA) between the lateral meniscus and the lateral tibial cartilage (Fig. 1A). The sensors were additionally secured against translational displacement during joint motion at the proximal tibia using small screws as previously described [33, 34]. Pre-tests ensured that the application of the pressure sensor did not alter the knee joint kinematics under any of the tested conditions.

Initially the knee joints were tested it the native state (Nat, Fig. 1A), followed by simulation of a total lateral meniscus meniscectomy (LMM, Fig. 1B) by arthroscopically detaching the anterior and posterior roots, the meniscotibial and meniscocapsular ligaments as well as the transverse intermeniscal ligament. The lateral meniscus was extracted through the anterior arthrotomy. Subsequently, the anterior and posterior transtibial tunnels were drilled to access the native lateral meniscus root insertions, similar to common MAT bone tunnel fixation drillings [6, 42]. During the drilling procedure, particular care was taken to avoid injuring the anterior cruciate ligament (ACL) insertions. 10 N graft pre-tensioning was conducted for both GT and ST autografts using a static weight which was held constant for 5 min [8]. First, the GT autograft was inserted through the anterior arthrotomy, while directly guiding the posterior anchoring suture through the respective tibial drill tunnel and fixing it via a traditional endobutton (Smith & Nephew, Andover, MA, USA). After passing the anterior suture of the GT autograft through the according drill hole, a slight force was applied to the anterior suture for graft reduction. Subsequently, the knee joint was flexed and extended several times to ensure an optimal fit of the graft before the anterior fixation was secured via an endobutton under the previously applied 10 N graft pre-tensioning. The autograft midbody was additionally sutured to the capsule in full knee extension using a single, vertical loop stitch to prevent meniscus extrusion [30]. In a final step, the GT autograft was removed, the drill holes were expanded to fit the size of the doubled ST autograft (ST, Fig. 1C), which was inserted and fixed similar to the GT technique. All surgical procedures were performed by an experienced arthroscopic surgeon, supported by an assistant surgeon. The knee joints were kept moist during preparation and testing by saline-saturated gauzes.

### Biomechanical testing

An established knee joint loading simulator [9] passively flexed and extended the knee joints for three cycles over 0°–120° degrees at a loading rate of 5°/sec and under an axial load of 200 N while applying four randomised loading conditions: (0) without moments; (ER) 1 Nm external rotation moment; (Val) 2.5 Nm valgus moment and (ERVal) combined 1 Nm external and 2.5 Nm valgus moment by applying dead weights on lever arms. These loading conditions have been identified to be kinematically sensitive to lateral meniscus alterations and, therefore, likely to have an impact on the lateral tibiofemoral contact mechanics [33]. Repeatable patellofemoral alignment was ensured by applying a dead weight of 10 N to the patellar tendon by means of a braided suture. The lateral tibiofemoral contact parameters (contact area (CA); peak contact pressure (CP_peak_), defined at 3 × 3 sensel checker area = 14.5 mm^2^; mean contact pressure (CP_mean_)) and the joint kinematics (internal/external rotations; varus/valgus rotations) were continuously recorded at a 5 Hz sampling rate. To account for the viscoelastic behaviour of the soft tissues, only the third flexion cycle of each test run was used for statistical evaluation.

### Statistical analysis

On the basis of a comparable study [21], an a priori sample size calculation (G*Power 3.1; [11]: *α* = 5%, Power (1-β) = 0.8,) resulted in *n* = 12. Due to slightly lower expected differences between the meniscus states and the feedback from the statistics department, the sample size was increased to *n* = 14. Valgus/Varus rotation, External/Internal rotation and the according contact pressure (CP_peak_, CP_mean_) automatically assessed at five defined flexion angles during the third flexion cycle (0°, 30°, 60°, 90°, 120°) using customised MATLAB routines (v. 2018b, The MathWorks, Inc., Natick, MA, USA). Shapiro–Wilk testing resulted in non-normally distributed results data. Accordingly, non-parametric statistical analyses were performed using a statistical software package (SPSS 25.0, SPSS Inc., IBM Company, Armonk, USA): Friedman testing was conducted to compare kinematic and pressure data of the different knee conditions (Nat vs. LMM vs. GT vs. ST) at the five flexion angles. Post hoc analysis with Bonferroni correction for multiple comparisons was performed when necessary. The level of significance was set to *p* < 0.05 for all statistical analyses.

## Results

### Joint kinematics

**Fig. 2:**
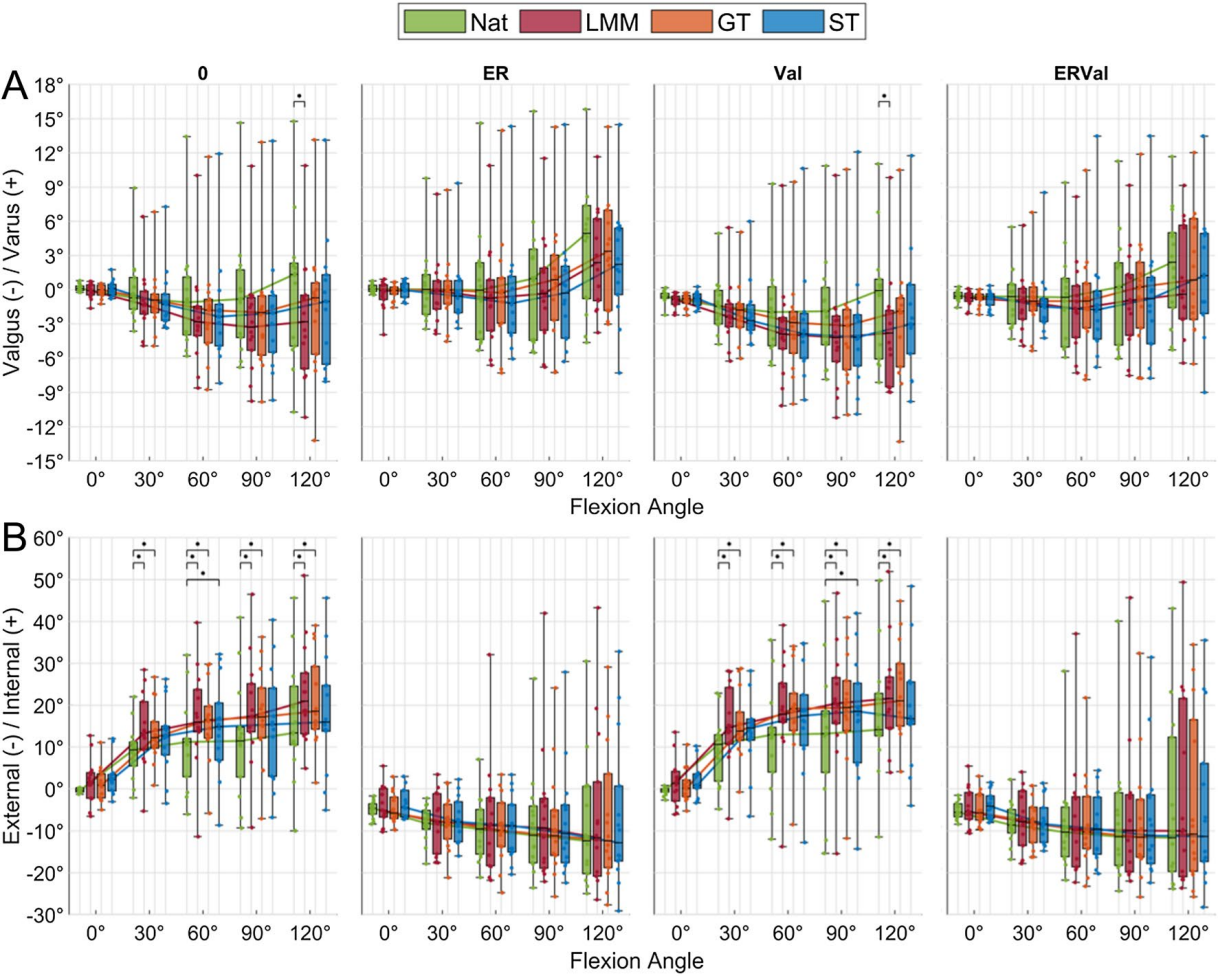
Box plots (minimum, maximum, median, 25th and 75th percentile values) of **A** valgus-varus and **B** external–internal rotations at five selected flexion angles (0°, 30°, 60°, 90°, 120°) and four knee conditions (Nat = native; LMM = (total) lateral meniscectomy; GT = gracilis tendon autograft reconstruction; ST = (doubled) semitendinosus tendon autograft reconstruction) under an axial load of 200 N and four different loading scenarios (0 = without external moments; ER = external rotation moment of 1 Nm; Val = valgus moment of 2.5 Nm; ERVal = combined external (1 Nm) and valgus (2.5 Nm) moment). Non-parametric statistical analyses: n = 14; * *p* < 0.05

Compared to the native condition, the other knee joint conditions (LMM, GT, ST) did not affect the varus-valgus rotation, except for the LMM state at 90° flexion (*p* < 0.01, Fig. 2). By contrast, internal rotation was significantly increased by up to 58% (*p* < 0.02) after LMM (Fig. 2). Following application of the GT autograft, the knee joints also indicated a significantly increased internal rotation ability when compared to the native joint condition in the loading scenarios without external loads (0, *p* < 0.03) and under 2.5 Nm valgus stress (Val, *p* < 0.01). By contrast, the ST autograft was able to restore rotational stability to the intact situation, except under 60° knee joint flexion without external moments (0, *p* < 0.04) and under 90° knee joint flexion with combined valgus stress (Val, *p* < 0.02). The here investigated knee joint conditions did not display altered knee joint kinematics under pure ER or combined ERVal (n.s.) loads.

### Lateral tibiofemoral contact mechanics

In the native knee joint, the lateral CP_peak_ ranged on average between 0.58 MPa (0) and 1.54 MPa (ERVal) (Table 1). Following GT autograft application, a significant decrease in the CP_peak_ compared to the LMM was only indicated in 30° and 60° knee joint flexion without the application of external moments (0, *p* < 0.04) and in 120° knee joint flexion under an external rotation moment application of 1 Nm (*p* < 0.02, ER). ST autograft implantation resulted in a significantly lower CP_peak_ when compared to the LMM state (− 37%, *p* < 0.02) for all observed test conditions and flexion angles (Table 1). The mean contact pressure CP_mean_ ranged from 0.19 MPa, observed in the ST state without external loads up to 0.71 MPa (+ 273%) observed in the LMM with ER loading (Table 2). The CP_mean_ values were always the highest in the LMM state. Furthermore, compared to the intact knee state, LMM revealed a significantly higher CP_mean_ for all loading scenarios (*p* < 0.04) except for the valgus state. Application of the ST autograft decreased the CP_mean_ to be comparable to the intact meniscus state for all loading situations. LMM resulted in a significantly decreased CA by up to 58% when compared to the intact meniscus condition (p < 0.01, Table 3). Both autograft procedures, GT and ST, were unable to restore the CA to that comparable to the intact meniscus state (Table 3).

**Table 1:** Minimum, median and maximum peak contact pressure (CP_peak_) values in MPa at five selected flexion angles (0°, 30°, 60°, 90°, 120°) and four knee conditions (Nat = native; LMM = (total) lateral meniscectomy; GT = gracilis tendon autograft reconstruction; ST = (doubled) semitendinosus tendon autograft reconstruction) under an axial load of 200 N and four different loading scenarios (0 = without external moments; ER = external rotation moment of 1 Nm; Val = valgus moment of 2.5 Nm; ERVal = combined external (1 Nm) and valgus (2.5 Nm) moment)

CP_peak_ in MPa		O				ER				Val				ERVal			
		Nat	LMM	GT	ST	Nat	LMM	GT	ST	Nat	LMM	GT	ST	Nat	LMM	GT	ST
0°	Max	1.92	2.20	1.74	2.22	1.92	2.02	1.85	1.81	1.81	1.52	1.78	1.42	2.86	2.41	2.03	2.04
	Med	0.73	0.81*	0.63	**0.53**	0.90	1.20	1.29	0.76	0.93	0.76	0.95	0.66	1.08	1.13	1.54	1.11
	Min	0.37	0.25	0.02	0.14	0.49	0.31	0.19	0.25	0.33	0.10	0.26	0.29	0.54	0.32	0.23	0.18
30°	Max	1.02	1.76	1.69	2.15	1.73	2.67	1.93	1.65	1.44	1.94	1.60	1.41	2.00	2.35	2.05	1.85
	Med	0.58	0.98*	**0.47**	**0.40**	**0.75**	1.49*	1.08	**0.77**	0.62	0.81	0.60	0.43	**0.97**	1.54*	1.21	**0.90**
	Min	0.16	0.25	0.10	0.22	0.19	0.28	0.31	0.16	0.22	0.22	0.27	0.13	0.33	0.31	0.19	0.15
60°	Max	1.49	1.88	1.44	1.77	2.04	1.67	2.03	1.58	2.03	1.91	1.75	1.42	2.41	2.29	2.04	1.86
	Med	0.76	0.84*	**0.46**	**0.36**	0.76	1.18	1.03	0.74	**0.82**	**1.02**	0.68	0.52*	**0.89**	1.46*	1.18*	**0.88**
	Min	0.08	0.37	0.21	0.19	0.04	0.31	0.34	0.19	0.15	0.22	0.37	0.28	0.20	0.34	0.31	0.23
90°	Max	1.93	1.52	1.75	1.54	1.69	2.09	1.81	1.57	2.44	2.93	1.90	1.83	2.12	2.48	1.95	1.85
	Med	**1.04**	**0.84**	0.56	0.42*	0.82	1.02*	0.77	**0.61**	**1.13**	**1.17**	0.92	0.63*	0.98	1.17*	0.94	**0.80**
	Min	0.20	0.58	0.27	0.18	0.16	0.25	0.17	0.26	0.36	0.18	0.54	0.17	0.19	0.45	0.18	0.32
120°	Max	2.11	3.53	2.85	1.93	1.77	2.02	1.83	1.46	2.97	3.73	4.08	1.97	2.11	2.24	1.96	1.58
	Med	1.09	1.18	1.16	0.62	0.77	1.05*	**0.75**	**0.53**	**1.32**	**1.44**	1.42	0.75*	1.01	1.34*	0.99	**0.72**
	Min	0.48	0.13	0.03	0.29	0.12	0.14	0.04	0.15	0.81	0.18	0.17	0.28	0.43	0.24	0.09	0.23

Statistically different to bold numbers within one loading condition and the according flexion angle

Non-parametric statistical analyses: *n* = 14; *p < 0.05

**Table 2:** Minimum, median and maximum mean contact pressure (CP_mean_) values in MPa at five selected flexion angles (0°, 30°, 60°, 90°, 120°) and four knee conditions (Nat = native; LMM = (total) lateral meniscectomy; GT = gracilis tendon autograft reconstruction; ST = (doubled) semitendinosus tendon autograft reconstruction) under an axial load of 200 N and four different loading scenarios (0 = without external moments; ER = external rotation moment of 1 Nm; Val = valgus moment of 2.5 Nm; ERVal = combined external (1 Nm) and valgus (2.5 Nm) moment)

CP_mean_ in MPa		O				ER				Val				ERVal			
		Nat	LMM	GT	ST	Nat	LMM	GT	ST	Nat	LMM	GT	ST	Nat	LMM	GT	**ST**
0°	Max	0.61	1.18	0.63	0.84	0.66	1.22	0.91	1.04	0.72	0.84	0.71	0.85	1.00	1.12	1.09	1.11
	Med	**0.30**	0.47*	0.41	0.31	**0.35**	0.62*	0.57	**0.43**	0.37	0.46	0.46	0.41	**0.38**	0.60*	0.59	0.53
	Min	0.19	0.15	0.05	0.10	0.24	0.23	0.12	0.14	0.16	0.11	0.18	0.19	0.22	0.32	0.13	0.13
30°	Max	0.39	1.11	0.73	0.66	0.60	1.37	0.83	0.96	0.46	0.83	0.61	0.65	0.70	1.26	0.92	1.04
	Med	**0.25**	0.44*	**0.26**	0.26	**0.31**	0.71*	**0.54**	**0.31**	0.28	0.45	0.36	0.29	**0.35**	0.69*	0.56	**0.44**
	Min	0.08	0.11	0.09	0.13	0.11	0.29	0.16	0.13	0.10	0.12	0.14	0.09	0.17	0.32	0.13	0.12
60°	Max	0.49	1.30	0.65	0.66	0.75	0.88	1.03	0.77	0.67	0.92	0.60	0.63	0.89	1.12	1.11	0.70
	Med	0.29	0.40	0.28	0.19	**0.31**	0.60*	0.45*	**0.38**	0.35	0.47	0.38	0.30	**0.37**	0.67*	0.57	**0.43**
	Min	0.06	0.24	0.12	0.12	0.06	0.27	0.18	0.12	0.08	0.12	0.19	0.15	0.11	0.32	0.20	0.16
90°	Max	0.61	0.79	0.59	0.63	0.51	0.83	0.68	0.71	0.71	1.23	0.78	0.60	0.12	0.22	0.11	0.17
	Med	0.32	0.47*	0.33	**0.19**	**0.32**	0.55*	0.38	0.31	0.42	0.58	0.47	0.32	**0.34**	0.54*	0.45	0.35
	Min	0.10	0.28	0.15	0.13	0.09	0.15	0.10	0.11	0.12	0.11	0.28	0.11	0.68	0.95	0.77	0.73
120°	Max	0.59	1.26	0.95	0.65	0.99	0.95	0.85	0.77	0.75	1.35	1.05	0.69	0.86	1.03	0.87	0.87
	Med	0.37	0.63*	0.43	**0.28**	**0.32**	0.51*	0.31	**0.27**	0.47	0.69*	0.54	**0.37**	0.40	0.58	0.43	0.40
	Min	0.24	0.15	0.06	0.20	0.08	0.10	0.06	0.12	0.30	0.11	0.21	0.13	0.17	0.14	0.08	0.12

Statistically different to bold numbers within one loading condition and the according flexion angle

Non-parametric statistical analyses: *n* = 14; *p < 0.05

**Table 3:** Minimum, median and maximum contact area (CA) values in mm^2^ at five selected flexion angles (0°, 30°, 60°, 90°, 120°) and four knee conditions (Nat = native; LMM = (total) lateral meniscectomy; GT = gracilis tendon autograft reconstruction; ST = (doubled) semitendinosus tendon autograft reconstruction) under an axial load of 200 N and four different loading scenarios (0 = without external moments; ER = external rotation moment of 1 Nm; Val = valgus moment of 2.5 Nm; ERVal = combined external (1 Nm) and valgus (2.5 Nm) moment)

CA in mm^2^		O				ER				Val				ERVal			
		Nat	LMM	GT	ST	Nat	LMM	GT	ST	Nat	LMM	GT	ST	Nat	LMM	GT	ST
0°	Max	457	274	314	340	526	225	314	211	541	237	312	322	525	212	280	338
	Med	282*	**113**	**107**	**99**	232*	**99**	**112**	**116**	263*	**117**	**137**	**108**	270*	**112**	**127**	147*
	Min	129	34	16	40	108	23	45	39	151	19	35	40	135	26	45	39
30°	Max	428	295	325	338	383	233	274	235	436	238	341	315	409	225	277	328
	Med	307*	**134**	163	158	259*	**105**	**138**	**164**	315*	**142**	184	170	299*	**113**	**142**	155
	Min	106	23	61	39	129	24	89	48	153	56	97	35	158	27	92	48
60°	Max	373	274	311	327	430	224	280	232	354	224	343	322	407	235	278	327
	Med	241*	**110**	155	157	255*	**95**	**115**	**98**	245*	**118**	163	151	258*	**107**	**142**	119
	Min	63	35	87	43	35	32	77	43	113	60	93	35	118	29	72	40
90°	Max	404	266	315	372	406	208	277	206	335	219	275	354	467	232	270	332
	Med	216*	**97**	136	159	299*	**111**	134	**100**	222*	**90**	137	145	311*	**119**	**166**	**126**
	Min	108	53	47	52	166	32	82	39	119	48	56	45	196	37	95	43
120°	Max	309	274	317	322	398	203	280	237	354	261	272	348	402	230	278	325
	Med	**189**	75*	120	**131**	282*	**105**	162	**95**	**222**	80*	126	**130**	270*	**108**	**139**	**114**
	Min	93	11	11	47	77	35	21	47	90	29	32	43	119	29	60	47

Statistically different to bold numbers within one loading condition and the according flexion angle

Non-parametric statistical analyses: *n* = 14; *p < 0.05

## Discussion

The most important finding of this biomechanical in vitro study is that the ST meniscus autograft was able to significantly improve both the joint kinematics and the tibiofemoral contact parameters after LMM. To the best of the authors’ knowledge, this is the first study investigating the kinematic knee joint changes and the impact on lateral tibiofemoral contact mechanics after total LMM and total meniscus replacement by a single-bundle GT and doubled ST autografts, which were surgically applied in the manner of a meniscus allograft transplant. The most important kinematic finding of the present study was that the LMM-induced rotational instability of the knee joint which was seen during the application of no external moments and during the application of 1 Nm valgus could be restored by the application of the doubled ST meniscus autograft, whereas application of the GT autograft indicated only a positive trend. With respect to the detrimental impact of the LMM on the lateral tibiofemoral contact mechanics, again, the ST autograft was able to restore the CP_mean_ comparable to the intact meniscus state. Further, CP_peak_ was significantly improved after ST autograft application compared with the LMM state. This was also seen for the CA, except for the external rotation loading condition. With regards to the tibiofemoral contact, the GT autograft application indicated only a trend towards contact mechanics improvement compared to the total meniscectomy state. In conclusion, the hypothesis that a ST meniscus leads to significantly improved biomechanics after LMM, could be corroborated.

In addition to the ACL, the posterior horn of the lateral meniscus is a main rotation stabiliser of the knee joint, particularly during deep knee flexion [27, 29]. LMM induced a significant rotational instability during all knee joint flexion in the knee joints, which was in accordance with the literature [12, 29]. The GT autograft failed in restoring the rotational stability, whereas the doubled ST autograft significantly improved the rotational stability in the 0 and Val loading conditions. Considerable morphological variations of both these tendons have been reported, with the ST being longer and indicating a larger mean cross-section compared to the GT [28]. The doubled ST meniscus autograft in this study had a larger cross-sectional area (26.7 ± 5.3 mm^2^) compared to the GT autograft (12.2 ± 3.4 mm^2^). The mean radial cross-section meniscus area is 34.7 ± 9.6 mm^2^ [4], indicating a better geometrical cross-section correlation of the doubled ST autograft. In silico analyses have predicted that the meniscus geometry and, particularly, its cross-sectional area considerably affects the meniscus kinematics and the related knee joint kinematics [23]. In vitro studies utilising lateral MAT indicated that an appropriate allograft size is able to restore the native knee kinematics [26]. In addition, Lee et al. reported in their study that the degree of lateral MAT size mismatching correlates with meniscus extrusion and associated knee laxity during motion [19]. Therefore, the results of the present study can be interpreted in a way that the doubled ST meniscus autograft with a more appropriate cross-section significantly improved the knee kinematics almost to the native situation, whereas the smaller GT graft was unable to achieve this. Moreover, an in vitro study on the effect of lateral MAT sizing revealed that a size mismatch of < 10% is acceptable, while greater deviations may lead to failure or subsequent development of degenerative changes [7]. This, therefore, supported the assumption that a single-bundle GT meniscus autograft might be too small to be able to restore knee joint kinematics after LMM.

**Fig. 3:**
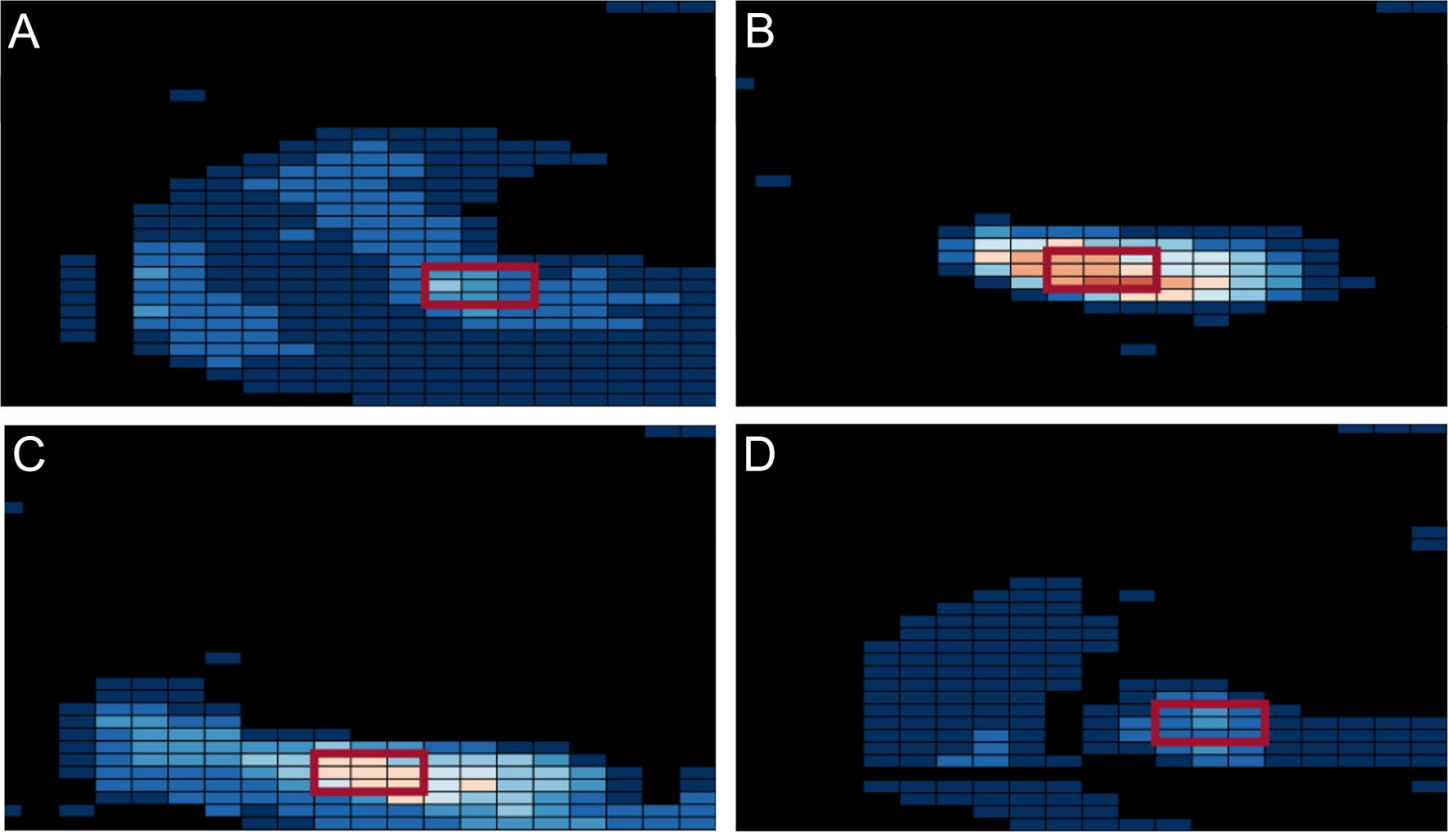
Representative peak contact pressure (CP_peak_) plots without the application of external moments and under 200 N axial loading at 60° knee flexion of the **A** native; **B** (total) lateral meniscectomy (LMM); **C** gracilis tendon autograft reconstruction (GT) and **D** doubled semitendinosus tendon autograft reconstruction (ST), indicating a shifting of the contact pressure from the homogenous contact in the native state towards the typical cartilage to cartilage contact during LMM to the posterior horn area of the autograft after GT and the more native-like contact after ST autograft

A recent review on the impact of different test setups and meniscal states on the tibiofemoral contact pressure indicated a major impact of the applied axial load on the CP_peak_ [38]. The CP_peak_ measurements of the present study were in the same range of those reported in the intact state and after performing a total LMM under an applied axial loading of 200 N [38]. Therefore, the observed differences between LMM and intact under 200 N axial loading were expectable and the tibiofemoral contact trends observed after autologous meniscus replacement of the present study were inevitable. However, it should be mentioned that application of physiological knee joint loadings would lead to more pronounced differences in CP_peak_ measurements [38]. Biomechanical and clinical MAT studies have indicated that using oversized lateral meniscus allografts leads to greater contact forces at the lateral compartment, while undersized ones results in greater forces across the meniscus body itself [7, 37]. This pattern was also observed in the case of GT autografts, resulting in a shifting of the cartilage-to-cartilage load in the LMM state (Fig. 3B) to the posterior horn of the GT autograft in 60° knee joint flexion (Fig. 3C). In conclusion, the current study findings are comparable to those achieved after lateral MAT procedures indicating a failure of undersized transplants and similar contact mechanics to intact knees when the grafts were within the range of a 10% size mismatch [7]. The previously mentioned loading shift of the CP_peak_ from the cartilage contact to the meniscus body might be an explanation for the constant CA when comparing the LMM and autograft states. However, even after application of the best possible size allograft, the CA is reduced [24], which is even more pronounced in the case of a non-anatomical reconstruction. A standardised autograft pre-tensioning was achieved at 10 N using a static weight which was attached to a special pre-tensioning clamp [8]. Prior to starting the flexion–extension cycles, the graft pre-tension was kept constant for five minutes. However, as indicated by von Lewinski et al. graft pre-tensioning significantly affected the CA at different knee joint flexion states. Unfortunately, even with the application of an ideal pre-tensioning force, the intact CA cannot be restored during MAT [40]. Therefore, the CA changes observed in the present study were expected to some extent.

Several limitations must be considered when interpreting the results of the present study. First, the inherent in vitro study design only reflects time zero evaluations and cannot account for in vivo changes postoperatively after an autograft implantation procedure. On the basis of recent clinical results utilising the same autografting procedure [30], it can be assumed that both the knee joint kinematics and the tibiofemoral load transfer will further improve during the remodelling phase of the autograft into a meniscus-like structure. Second, the anteroposterior length of the autologous grafts to ensure complete size matching as it is recommended for MAT procedure was not assessed [35]. Latest developments in three-dimensional allograft sizing using the closest mean surface distance method that is based on 3D-MRI sizing with the contralateral meniscus have shown, that all dimensions of the graft sizing must be considered to potentially improve the mid- and long-term survivorships of MAT procedures [1]. Therefore, care should be taken in future studies to achieve also a best possible anteroposterior length match when proceeding the autologous doubled ST meniscus autograft. Third, the autografts were secured against extrusion using only one single, vertical loop stitch at the midbody of the graft. While this single fixation seems to have been reasonable for the present biomechanical investigation under limited 200 N axial loading, in the clinical situation more suture stiches (e.g. spaced 10 mm apart along the whole circumferential length) would be required, as performed in the clinical meniscus autograft study by Rönnblad et al. [30] or other MAT studies [7]. Fourth, several soft tissues were removed and a horizontal arthrotomy was performed to insert the pressure sensor, potentially affecting both, kinematics and the load transfer of the knee joint. The impact of the consecutive preparation steps on our target parameters were extensively elaborated during pre-tests, with no significant impact on the here investigated outcome measures. Furthermore, the loads applied to the cadaveric specimens in this study did not reflect those arising during daily activities. However, based on our own experience from earlier studies [33, 34], particular loading scenarios were chosen for the present study, including external rotation and valgus moments, with the aim to have potentially the greatest impact on lateral meniscus biomechanics. The main technical challenge was the placement of the pressure sensors on the tibial plateau to obtain accurate measurements throughout the investigated full knee joint flexion and extension range of 0°–120°. The pressure sensor was anteriorly secured against displacement, ensuring the ability to adequately measure the tibiofemoral contact mechanics in the extension to mid-flexion states (≤ 60°). However, particularly in large-sized male knees, it might not have been possible to obtain some pressure measurement data in the deep flexion states.

In the context of clinical applicability, the present biomechanical results need to be interpreted with care, because both, remodelling and failure mechanisms cannot be investigated by such an in vitro study. However, small animal [13], large animal [17] and pilot clinical studies [17, 30] on meniscus tendon autograft substitution indicated a secondary remodelling of the transplanted tendons towards wedge-like meniscus-shaped tissue. The first clinical study on 20 patients were performed in 1989 by Kohn, where the medial meniscus was replaced by a part of the quadriceps tendon [17]. A 1-year follow-up arthroscopy indicated an unaltered articular cartilage, clinical improvement and autograft integration. Johnson et al. [14] performed a pilot study on five patients with pre-existing lateral OA. During their 9-24-month follow-up, a partial physical integration of the grafts was observed without joint surface preservation. The positive results from Kohn were then reinforced by a recent clinical study from Rönnblad et al. [30] who included seven patients and performed also a 1-year follow-up. They indicated the potential of doubled ST autografts for meniscus-like structure remodelling and clinical improvement regarding IKDC, KOOS and the Lysholm scores. The results of the present study again supported these short-term clinical findings, also from a biomechanical point of view. Should these overall positive outcomes be further reinforced by studies with a higher level of evidence, the ST meniscus autograft might be a promising alternative for young patients who require a total meniscus replacement after a traumatic meniscus injury without any signs of OA. In case the articular cartilage is already compromised too greatly by degeneration a meniscus replacement is unlikely to prevent the progression of OA [30]. Therefore, proper patient selection is key for this procedure to ensure the best possible patient-related outcome measures. In conclusion, further studies are required to finally prove that a doubled ST meniscus autograft is a viable alternative for an artificial total meniscus replacement or MAT.

## Conclusion

The doubled ST lateral meniscus autograft improved the knee joint kinematics significantly and restored the tibiofemoral contact mechanics almost comparable to the native situation. Thus, from a biomechanical point of view, ST meniscus autografts might be a potential treatment alternative for patients who are indicated for meniscus allograft transplantation.

## Supplementary Information

Below is the link to the electronic supplementary material.

Supplementary file1 (XLSX 35 KB)

Supplementary file2 (XLSX 35 KB)

Supplementary file3 (XLSX 27 KB)

Supplementary file4 (XLSX 34 KB)

Supplementary file5 (XLSX 34 KB)

Supplementary file6 (PDF 529 KB)

Supplementary file7 (DOCX 21 KB)

Supplementary file8 (DOCX 21 KB)

## Data Availability

All data generated or analysed during this study are included in this published article (and its supplementary information files).

## Abbreviations

0: Test condition without external moments
ACL: Anterior cruciate ligament
CA: Contact area in mm^2^
CP_mean_: Mean tibiofemoral contact pressure in MPa
CP_peak_: Peak tibiofemoral contact pressure in MPa defined at a 3 × 3 sensel checker area of 14.5 mm^2^
ER: External rotation moment (1 Nm)
ERVal: Combined external and valgus rotation moment (1 Nm + 2.5 Nm)
GT: Gracilis tendon
LMM: (Total) Lateral meniscectomy
MAT: Meniscus allograft transplant
Nat: Native knee joint
OA: Osteoarthritis
ST: (Doubled) Semitendinosus tendon
Val: Valgus rotation moment (2.5 Nm)
