# Supplementary material for: Autologous semitendinosus meniscus graft significantly improves knee joint kinematics and the tibiofemoral contact after complete lateral meniscectomy
Source: Knee Surg Sports Traumatol Arthrosc. 2023 Jan 5;31(7):2956–65. doi: 10.1007/s00167-022-07300-z (PMC10276070; doi:10.1007/s00167-022-07300-z)
Supplement: Supplementary file 6 — Supplementary file6 (PDF 529 KB) [file 167_2022_7300_MOESM6_ESM.pdf]

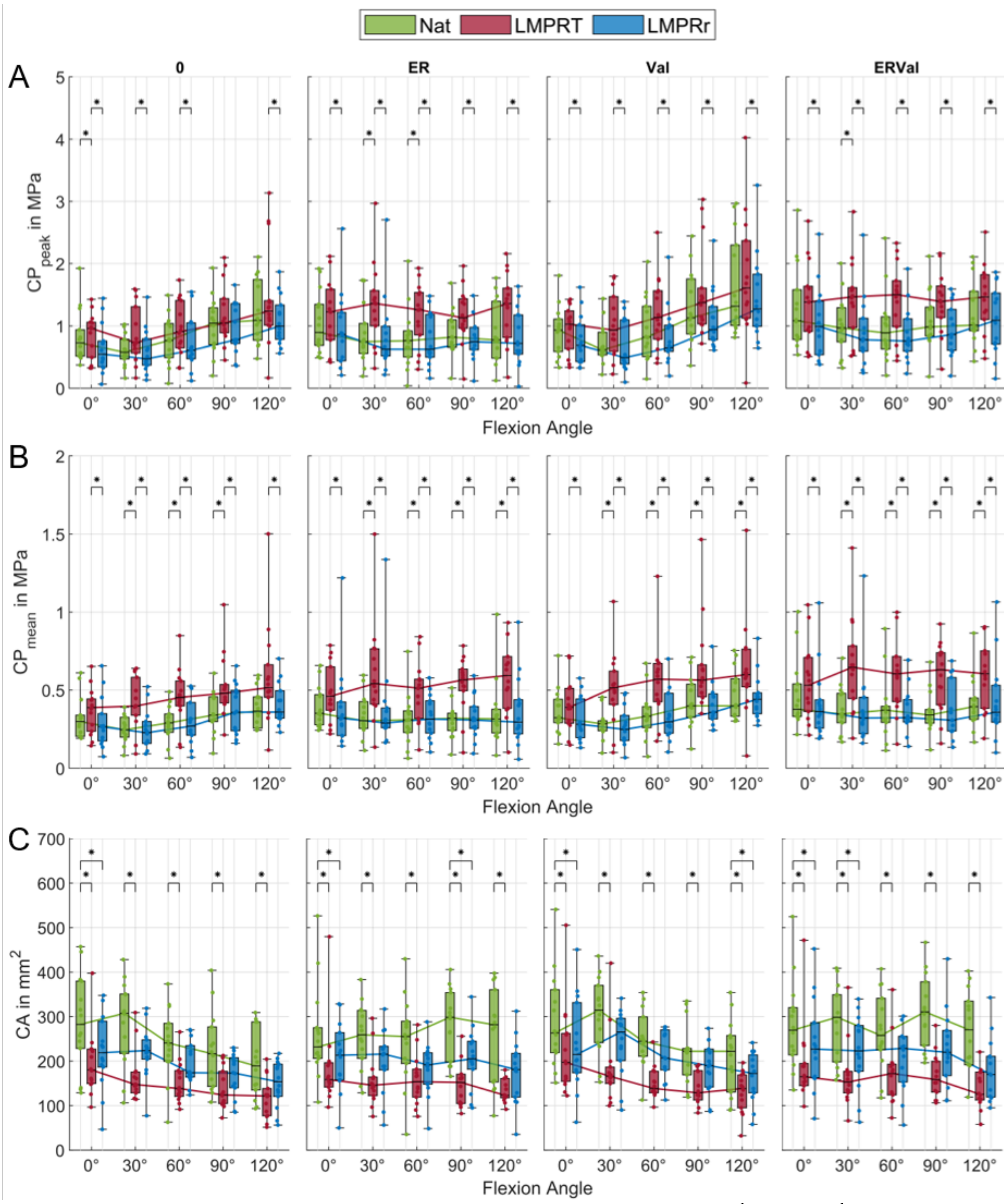

Supplementary Fig.1: Box plots (minimum, maximum, median, 25th and 75th percentiles) of (A) peak contact pressures (CP_{peak}) in MPa, (B) mean contact pressures (CP_{mean}) in MPa and (C) contact area (CA) in mm² at five selected flexion angles (0°, 30°, 60°, 90°, 120°) and four knee conditions (Nat = native; LMM = (total) lateral meniscectomy; GT = gracilis tendon autograft reconstruction; ST = (doubled) semitendinosus tendon autograft reconstruction) under an axial load of 200 N and four different loading scenarios (0 = without external moments; ER = external rotation moment of 1 Nm; Val = valgus moment of 2.5 Nm; ERVal = combined external (1 Nm) and valgus (2.5 Nm) moment). Non-parametric statistical analyses: n = 14; *$p < 0.05$.
