# Supplementary material for: Autologous semitendinosus meniscus graft significantly improves knee joint kinematics and the tibiofemoral contact after complete lateral meniscectomy
Source: Knee Surg Sports Traumatol Arthrosc. 2023 Jan 5;31(7):2956–65. doi: 10.1007/s00167-022-07300-z (PMC10276070; doi:10.1007/s00167-022-07300-z)
Supplement: Supplementary file 7 — Supplementary file7 (DOCX 21 KB) [file 167_2022_7300_MOESM7_ESM.docx]

Supplementary Table 1: Minimum, median and maximum valgus (-) /varus (+) rotations in ° at five selected flexion angles (0°, 30°, 60°, 90°, 120°) and four knee conditions (Nat = native; LMM = (total) lateral meniscectomy; GT = gracilis tendon autograft reconstruction; ST = (doubled) semitendinosus tendon autograft reconstruction) under an axial load of 200 N and four different loading scenarios (0 = without external moments; ER = external rotation moment of 1 Nm; Val = valgus moment of 2.5 Nm; ERVal = combined external (1 Nm) and valgus (2.5 Nm) moment). Non-parametric statistical analyses: n = 14; ***p < 0.05**.

| **Varus/Valgus in °** | | **O** | | | | **ER** | | | | **Val** | | | | **ERVal** | | | |
| --- | --- | --- | --- | --- | --- | --- | --- | --- | --- | --- | --- | --- | --- | --- | --- | --- | --- |
|  |  | **Nat** | **LMM** | **GT** | **ST** | **Nat** | **LMM** | **GT** | **ST** | **Nat** | **LMM** | **GT** | **ST** | **Nat** | **LMM** | **GT** | **ST** |
| **0°** | Max | +0.79 | +0.73 | +0.55 | +1.76 | +0.77 | +0.92 | +0.67 | +0.97 | +0.17 | -0.24 | -0.27 | +0.26 | +0.27 | +0.09 | +0.03 | +0.35 |
|  | **Med** | +0.11 | -0.08 | 0.00 | +0.12 | +0.06 | -0.08 | -0.06 | -0.04 | -0.60 | -0.86 | -0.77 | -0.75 | -0.57 | -0.71 | -0.69 | -0.57 |
|  | Min | -0.23 | -1.63 | -1.26 | -0.80 | -0.46 | -3.95 | -1.57 | -1.20 | -2.23 | -2.22 | -2.25 | -1.93 | -1.57 | -2.11 | -2.19 | -2.24 |
| **30°** | Max | +8.93 | +6.41 | +6.83 | +7.28 | +9.78 | +8.39 | +8.75 | +9.35 | +4.94 | +5.45 | +5.05 | +5.99 | +5.50 | +5.65 | +6.78 | +8.53 |
|  | **Med** | -0.72 | -1.30 | -0.91 | -1.29 | +0.03 | -0.09 | -0.02 | -0.56 | -1.46 | -2.40 | -1.79 | -2.71 | -0.60 | -1.02 | -1.01 | -1.50 |
|  | Min | -3.64 | -4.92 | -4.94 | -3.37 | -3.46 | -3.88 | -4.54 | -3.39 | -4.78 | -6.26 | -6.03 | -4.84 | -4.33 | -4.93 | -5.36 | -4.24 |
| **60°** | Max | +13.43 | +10.03 | +11.66 | +11.93 | +14.60 | +10.90 | +13.97 | +14.32 | +9.29 | +9.14 | +9.46 | +10.64 | +9.39 | +8.16 | +10.49 | +13.47 |
|  | **Med** | -1.11 | -2.82 | -1.80 | -2.37 | -0.03 | -0.74 | -0.27 | -1.19 | -1.97 | -3.90 | -2.89 | -3.89 | -0.67 | -1.68 | -1.00 | -1.77 |
|  | Min | -5.82 | -8.62 | -8.76 | -8.20 | -5.32 | -6.62 | -7.30 | -6.07 | -6.63 | -10.17 | -10.01 | -9.66 | -5.93 | -7.30 | -7.88 | -6.79 |
| **90°** | Max | +14.63 | +10.85 | +12.93 | +13.05 | +15.64 | +11.53 | +14.27 | +14.48 | +10.87 | +10.04 | +10.56 | +12.08 | +11.28 | +9.16 | +11.89 | +13.48 |
|  | **Med** | -0.82 | -3.29 | -2.01 | -2.06 | +0.97 | -0.34 | +0.87 | -0.29 | -1.90 | -4.19 | -3.16 | -4.17 | +0.23 | -0.96 | +0.25 | -0.79 |
|  | Min | -6.80 | -9.77 | -9.83 | -9.68 | -5.53 | -6.78 | -7.26 | -6.30 | -7.87 | -11.20 | -10.96 | -10.89 | -6.05 | -7.53 | -7.76 | -7.75 |
| **120°** | Max | +14.77 | +10.89 | +13.15 | +13.12 | +15.82 | +11.66 | +14.28 | +14.47 | +11.05 | +9.84 | +10.51 | +11.76 | +11.68 | +9.15 | +12.03 | +13.47 |
|  | **Med** | **+1.34*** | **-2.82** | -0.71 | -1.03 | +4.95 | +2.38 | +3.38 | +2.23 | **-0.07*** | **-3.80** | -1.89 | -3.02 | +2.41 | -0.40 | +0.85 | +1.23 |
|  | Min | -10.71 | -11.17 | -13.21 | -8.05 | -4.63 | -3.76 | -3.03 | -7.30 | -8.12 | -8.98 | -13.31 | -9.80 | -5.28 | -6.43 | -6.51 | -9.00 |
