# Supplementary material for: Autologous semitendinosus meniscus graft significantly improves knee joint kinematics and the tibiofemoral contact after complete lateral meniscectomy
Source: Knee Surg Sports Traumatol Arthrosc. 2023 Jan 5;31(7):2956–65. doi: 10.1007/s00167-022-07300-z (PMC10276070; doi:10.1007/s00167-022-07300-z)
Supplement: Supplementary file 8 — Supplementary file8 (DOCX 21 KB) [file 167_2022_7300_MOESM8_ESM.docx]

Supplementary Table 2: Minimum, median and maximum external (-) and internal (+) rotations in ° at five selected flexion angles (0°, 30°, 60°, 90°, 120°) and four knee conditions (Nat = native; LMM = (total) lateral meniscectomy; GT = gracilis tendon autograft reconstruction; ST = (doubled) semitendinosus tendon autograft reconstruction) under an axial load of 200 N and four different loading scenarios (0 = without external moments; ER = external rotation moment of 1 Nm; Val = valgus moment of 2.5 Nm; ERVal = combined external (1 Nm) and valgus (2.5 Nm) moment). Non-parametric statistical analyses: n = 14; ***p < 0.05**.

| **External / Internal in °** | | **O** | | | | **ER** | | | | **Val** | | | | **ERVal** | | | |
| --- | --- | --- | --- | --- | --- | --- | --- | --- | --- | --- | --- | --- | --- | --- | --- | --- | --- |
|  |  | **Nat** | **LMM** | **GT** | **ST** | **Nat** | **LMM** | **GT** | **ST** | **Nat** | **LMM** | **GT** | **ST** | **Nat** | **LMM** | **GT** | **ST** |
| **0°** | Max | +0.27 | +12.75 | +11.08 | +12.00 | -1.71 | +5.44 | +2.89 | +2.94 | +0.78 | +13.57 | +10.59 | +10.24 | -1.56 | +5.41 | +3.02 | +1.52 |
|  | **Med** | -0.43 | +1.49 | +0.81 | +2.81 | -4.98 | -4.09 | -4.79 | -3.61 | -0.36 | +1.31 | +0.08 | +0.80 | -5.59 | -5.44 | -5.74 | -4.14 |
|  | Min | -1.29 | -6.55 | -5.02 | -3.07 | -8.41 | -10.20 | -9.78 | -7.24 | -2.69 | -6.10 | -5.29 | -3.36 | -8.45 | -10.54 | -9.70 | -8.05 |
| **30°** | Max | +22.00 | +28.47 | +26.72 | +26.23 | -2.26 | +3.42 | +1.45 | +2.24 | +22.86 | +28.16 | +28.76 | +28.21 | +2.21 | +4.01 | +0.73 | +1.34 |
|  | **Med** | **+9.31*** | **+13.34** | **+12.25** | +12.65 | -8.29 | -7.82 | -8.08 | -7.43 | **+10.62*** | **+14.69** | **+13.83** | +14.56 | -8.66 | -7.86 | -7.94 | -8.41 |
|  | Min | -2.15 | -5.32 | +0.82 | -3.49 | -17.85 | -17.59 | -21.17 | -15.97 | -4.84 | -7.14 | -0.77 | -6.57 | -16.79 | -17.79 | -16.25 | -14.77 |
| **60°** | Max | +31.97 | +39.74 | +29.82 | +32.18 | +7.02 | +32.07 | +3.39 | +3.36 | +35.58 | +39.14 | +34.10 | +34.79 | +28.14 | +37.08 | +21.75 | +4.39 |
|  | **Med** | **+11.21*** | **+15.96** | **+16.54** | **+14.89** | -9.65 | -7.26 | -9.69 | -9.09 | **+12.96*** | **+17.85** | **+19.20** | +17.54 | -10.28 | -9.67 | -10.22 | -9.58 |
|  | Min | -6.06 | -11.35 | -5.85 | -8.68 | -21.11 | -21.79 | -24.76 | -20.39 | -11.96 | -13.74 | -8.53 | -12.74 | -21.76 | -22.29 | -23.22 | -20.20 |
| **90°** | Max | +40.96 | +46.52 | +36.30 | +40.39 | +26.34 | +41.99 | +24.11 | +27.90 | +44.85 | +46.80 | +40.97 | +41.97 | +40.09 | +45.71 | +32.39 | +31.44 |
|  | **Med** | **+11.46*** | **+17.35** | **+17.22** | +15.42 | -8.67 | -6.63 | -8.29 | -7.70 | **+13.19*** | **+20.41** | **+19.47** | **+18.57** | -11.36 | -9.90 | -11.48 | -11.09 |
|  | Min | -9.33 | -9.22 | -7.11 | -6.83 | -23.59 | -22.10 | -25.70 | -22.41 | -15.35 | -15.41 | -11.41 | -14.28 | -24.36 | -21.98 | -25.79 | -22.39 |
| **120°** | Max | +45.64 | +50.95 | +39.07 | +45.64 | +30.49 | +43.30 | +29.09 | +32.84 | +49.80 | +51.91 | +44.90 | +48.44 | +43.13 | +49.41 | +34.46 | +35.52 |
|  | **Med** | **+13.39*** | **+20.95** | **+18.59** | +16.00 | -8.79 | -6.11 | -7.13 | -6.46 | **+14.17*** | **+21.58** | **+21.03** | +16.83 | -11.62 | -9.99 | -10.74 | -11.30 |
|  | Min | -9.97 | +4.85 | +1.44 | -5.08 | -24.97 | -26.43 | -27.67 | -29.11 | -11.42 | +3.89 | +4.10 | -4.03 | -23.83 | -23.65 | -25.73 | -28.24 |
